# Antinociceptive and Anxiolytic and Sedative Effects of Methanol Extract of *Anisomeles indica*: An Experimental Assessment in Mice and Computer Aided Models

**DOI:** 10.3389/fphar.2018.00246

**Published:** 2018-04-12

**Authors:** Md. Josim Uddin, A. S. M. Ali Reza, Md. Abdullah-Al-Mamun, Mohammad S. H. Kabir, Mst. Samima Nasrin, Sharmin Akhter, Md. Saiful Islam Arman, Md. Atiar Rahman

**Affiliations:** ^1^Department of Pharmacy, Faculty of Science and Engineering, International Islamic University Chittagong, Chittagong, Bangladesh; ^2^Department of Applied Nutrition and Food Technology, Islamic University, Kushtia, Bangladesh; ^3^Department of Pharmacy, University of Rajshahi, Rajshahi, Bangladesh; ^4^Department of Biochemistry and Molecular Biology, University of Chittagong, Chittagong, Bangladesh

**Keywords:** *Anisomeles indica*, writhing test, central pain, peripheral pain, neuropharmacology

## Abstract

*Anisomeles indica* (L.) kuntze is widely used in folk medicine against various disorders including allergy, sores, inflammation, and fever. This research investigated the antinociceptive, anxiolytic and sedative effects of *A. indica* methanol extract. The antinociceptive activity was assessed with the acetic acid-induced writhing test and formalin-induced flicking test while sedative effects with open field and hole cross tests and anxiolytic effects with elevated plus maze (EPM) and thiopental-induced sleeping time tests were assayed. Computer aided (pass prediction, docking) analyses were undertaken to find out the best-fit phytoconstituent of total 14 isolated compounds of this plant for aforesaid effects. Acetic acid treated mice taking different concentrations of extract (50, 100, and 200 mg/kg, intraperitoneal) displayed reduced the writhing number. In the formalin-induced test, extract minimized the paw licking time of mice during the first phase and the second phase significantly. The open field and hole-cross tests were noticed with a dose-dependent reduction of locomotor activity. The EPM test demonstrated an increase of time spent percentage in open arms. Methanol extract potentiated the effect of thiopental-induced hypnosis in lesser extent comparing with Diazepam. The results may account for the use of *A. indica* as an alternative treatment of antinociception and neuropharmacological abnormalities with further intensive studies. The compound, 3,4-dihydroxybenzoic acid was found to be most effective in computer aided models.

## Introduction

In the last few decades ethnobotanical research has reflected multifarious medicinal properties of plants including analgesic (Wirth et al., 2005), anti-inflammatory (Kumar et al., 2013), anti-rheumatic (Kaur et al., 2012), anti-cancer (Fridlender et al., 2015), and anti-depressive activities (Sarris et al., 2011). A number of herbal preparations is prescribed as analgesic and anti-depressive in the literature of alternative medicine. Search for new analgesic and anti-depressive drugs from a wider hub of medicinally important plants has been more focused since the last couple of decades. This is due to the exploration of novel therapeutic agents for suppression or relief of pain as well as depression (Anil, 2010).

The emotional responses or unusual sensory linked with potential tissue damage contributed by muscle spasm, tumor, nerve damage, inflammation, exposure to noxious chemical, thermal or mechanical stimuli refer to pain (Morrison and Morrison, 2006). As a therapeutic option, non-steroidal anti-inflammatory drugs (NSAIDs) are chosen for mild to moderate pain, while steroidal and opioids for intense acute and severe pain conditions. However, the side effects of both NSAIDs and opioids limit their frequent and free usages (Grosser et al., 2011; Yaksh and Wallace, 2011). Anxiety which has been reported as a psychological disorder is described as an unpleasant emotional state for which the cause is neither readily identified nor perceived to be uncontrollable. Anxiety impairs performances and it is associated with numbers of medically unexplained symptoms triggering insomnia. It is independently and strongly associated with chronic illnesses and low levels of life-quality (Hoffman et al., 2008). Therapeutic agents available for the treatment of anxiety include benzodiazepines, opioids, and non-steroidal anti-inflammatory drugs. These agents, however, are not without significant side effects that limit their use. Tolerance and dependence to some of these agents particularly opioids and benzodiazepines, make the search for potent alternatives very important (Kimiskidis et al., 2007). Medicinal plants have contributed enormously to the development of important therapeutic drugs for modern medical sciences (Olorunnisola et al., 2012). There is an increasing recognition that medicinal plants might provide a viable source for new drug molecules, especially in the case of failure of the more popular synthetic drugs (Prasad et al., 2005).

*Anisomeles indica* belongs to Lamiaceae family. It is usually familiar as gobura of annual shrub class distributed in most of the districts of Bangladesh. *A. indica* leaves are used for children's whooping cough and fever (Yusuf et al., 1994; Rahman et al., 2007). The roots have long been used for allergy, uterine infection, sores, and mouth abscess. Root also has anti-inflammatory, astringent, tonic properties (Yusuf et al., 1994). The extract of this plant is found to work on inflammatory mediators, bacteria, tumor cell proliferation and melanogenesis (Wang and Huang, 2005; Hsieh et al., 2008; Rao et al., 2009; Huang et al., 2012). Water extract of *A. indica* has primarily been found as centrally working analgesic (Dharmasiri et al., 2003). We thus comprehensively evaluated the antinociceptive, anxiolytic, and sedative effects effects of *A. indica* methanol extract (MEAI) in mice model elucidating the structure-activity relationship for aforesaid biological activities with the isolated phytocompounds of this plant by using *in silico* PASS prediction and molecular docking tools.

## Methods and materials

### Plant sample

Whole plant material of *A. indica* (L.) Kuntze (Lamiaceae) was collected from the Rajshahi University premises, Bangladesh in September, 2012 by the authors, and plant sample was identified by an expert taxonomist Dr. Sheikh Bokhtear Uddin who is working in the Department of Botany of University of Chittagong. A voucher specimen (accession no. 1304) has been preserved in the institutional herbarium of the aforesaid Department.

### Extract preparation

The shade-dried whole plants of *A. indica* were powdered (500 g) to macerate in absolute methanol (purity 99.99%, 1,500 ml). Powdered material was placed into an amber bottle for a 7-days-exhaustive extraction with occasional stirring and shaking in every 3 days. The extracts obtained were pooled and filtered using Whatman Filter paper #1. The final combined methanol specimen (850 ml) was evaporated to dryness using a vacuum rotary evaporator (RE200 Biby Sterling, UK) and weighted (16.19 g dry weight, 3.23% w/W) to determine the yield of soluble constituents. The semi-solid black-green crude extract soluble in methanol was preserved at 4°C.

### Maintenance of experimental animals

Six-seven weeks old Swiss albino mice of both sexes (50% male and 50% female) weighing ~35–40 g were procured from the animal research division of the International Centre for Diarrheal Disease and Research, Bangladesh (ICDDR, B). Mice were housed in polycarbonated cages ensuring a standard laboratory condition of temperature 25°C and humidity 55–56% in a 12 h day light cycle. They had a free access to supplied pellet animal diet and tap water. Animal handling protocol was endorsed by the Planning and Development (P&D) committee of the Department of Pharmacy of International Islamic University Chittagong, Bangladesh.

### Drugs and reagents

Reagent grade formalin and acetic acid were purchased from MERCK, India through Taj Scientific Ltd. Diazepam and diclofenac-Na were brought from Sigma-Aldrich, USA via local supplier. Morphine sulfate kindly donated by Popular Pharmaceuticals Ltd., Bangladesh. All other reagents were of analytical grade unless otherwise specified.

### Antinociceptive activities

#### Acetic acid-induced writhing test

Either sex of mice (*n* = 6) weighing 35–40 g were divided into five groups. Normal control group received normal saline (10 ml/kg bw), reference control group received standard drug diclofenac sodium (10 mg/kg bw) while the rest of the groups were injected intraperitoneally with 50, 100, and 200 mg/kg bw of methanol extract of *A. indica* (MEAI). After 30 min of administration, the animals were injected (i.p.) 1% (v/v) (10 ml/kg bw) acetic acid. After 5 min of acetic acid injection, abdominal constrictions were counted for 10 min and the responses were compared with control group (Koster et al., 1959). Antinociceptive activity was calculated as the writhing percentage of inhibition. The percentage of inhibition was calculated using the following ratio:

$$\begin{array}{l} \text{Writhing inhibition}=\frac{\text{Mean no}.\text{ of writhing }\left(\text{control}\right)-\text{Mean no}.\text{ of writhing }\left(\text{test}\right)}{\text{Mean number of writhing }\left(\text{control}\right)}\times 100 \end{array}$$

#### Formalin induced licking test

Formalin induced biphasic method employed in mice model was assessed as described previously (Burgos et al., 2010). Formalin solution (2.5%, 20 μl) prepared by 0.9% saline solution was injected into the sub-plantar region of the right hind paw of mice. Animals were intraperitoneally pretreated with saline solution, morphine sulfate (10 mg/kg bw), diclofenac sodium (10 mg/kg bw) and MEAI (50, 100, and 200 mg/kgbw) 1 h prior to formalin injection. Pain response was measured by the licking and biting of the injected paw. Responses measured for 5 min is considered as first phase and 15–30 min is considered as second phase after formalin injection. First phase and second phase response corresponds to the neurogenic and inflammatory pain responses, respectively. Antinociceptive activity was calculated as the percentage inhibition of licking time.

$$\begin{array}{l} \left(\left(\%\right)\right)\text{ of inhibition}=\frac{\text{Mean of licking time }\left(\text{control}\right)-\text{Mean of licking time }\left(\text{test}\right)}{\text{Mean of licking time }\left(\text{control}\right)}\times 100 \end{array}$$

### Locomotor activity

#### Open field test

The spontaneous locomotor performances of MEAI were assessed by using the open field test as described earlier. Briefly, the mice were placed in the test room at least 1 h before each test for habituation. The open field devices were comprised of a Plexiglas square box (50 × 50 × 40 cm) with the floor divided into 25 small squares of equal dimensions (10 cm × 10 cm) marked by black lines. In this study, test animals were randomly divided into five groups. Normal control received orally 1% Tween-80 in water (10 ml/kg), positive control Diazepam (1 mg/kg) administered intraperitoneally and treatment groups received MEAI orally at the doses of 100, 200, and 400 mg/kg bw. One hour administration, each animal was placed individually at the center of the device and observed for 5 min to count the number of squares crossed by the animal with its four paws. The open field arena was thoroughly cleaned between each test so that the animal was not influenced by the odors of urine and feces of the previous one (Saleem et al., 2011). Inhibition of movements was calculated using the following formula:

$$\begin{array}{l} \text{Movements inhibition }\left(\left(\%\right)\right)=\frac{\text{Mean no}.\text{ of movements }\left(\text{control}\right)-\text{Mean no}.\text{ of movements }\left(\text{test}\right)}{\text{Mean number of movements }\left(\text{control}\right)}\times 100 \end{array}$$

### Hole cross test

Hole cross test was accomplished with slight modification of the protocol previously described by Takagi et al. (1971). Briefly, mice were randomly divided into five groups: Control group received orally 1% Tween-80 in water (10 ml/kg bw), positive control group Diazepam (1 mg/kg bw) administered intraperitoneally and treatment group received MEAI orally at the doses of 100, 200, and 400 mg/kg bw. Afterwards the animals were placed in a compartment where a steel partition was fixed in the middle having a size of 30 × 20 × 14 cm^3^. A 3 cm (diameter) hole was made at a height of 7.5 cm in the center of the cage. After administration of control, positive control and different concentrations of extract the animals were allowed to cross the hole from one chamber to another and the numbers of passage through the hole from one chamber to other was counted. The total number of passage was counted for a period of 5 min on 0, 30, 60, 90, and 120 min during the study period. Percentage inhibition of movements was calculated using the same formula used in open field test.

### Anxiolytic activity

#### Elevated plus-maze test (EPM)

The Elevated plus-maze test is the modified method of the validated assay of Lister for mice (Pellow and File, 1986). The apparatus consists of two open arms (35 × 5 cm^2^) and two closed arms (30 × 5 × 15 cm^3^) extended from a common central platform (5 × 5 cm^2^). The walls and floor of the closed arms are made of wood and painted black. The whole maze is elevated to a height of 50 cm above the basement. An edge (0.25 cm) was included around the perimeter of the open to facilitate exploration. Mice (35–40 g) were housed for 10 days prior to the test in the apparatus. To reduce stress, the mice were handled by the researcher on alternate days. Control group received orally 1% Tween-80 in water (10 ml/kg bw), positive control group Diazepam (1 mg/kg bw) administered intraperitoneally and treatment group received MEAI orally at the doses of 100, 200, and 400 mg/kg bw. After 30 min treatment with control, diazepam, and treatment group each mouse was set onto the center of the maze facing one of the enclosed arms. Number of entries and time spent onto the open arm were noted for a 5 min session. Throughout the test procedure a calm and smooth environment was ensured to obtain accurate results.

### Sedative activity

#### Thiopental sodium induced sleeping time test

In this test animals were divided into four groups comprised of six mice each. Vehicle (1% Tween-80 in water 10 ml/kg), diazepam (1 mg/kg bw) and MEAI (100, 200, and 400 mg/kg bw) were injected intraperitoneally into control group, reference group and test groups, respectively. After 20 mins of treatment, thiopental sodium (40 mg/kg bw) was injected to each mouse for inducing sleep. The animals were observed to record the time between thiopental sodium administrations to loss of righting reflex (latent period) and duration between the loss and regaining of righting reflex (sleep duration) (File and Pellow, 1985). Percentage of effect was calculated using the following formula:

$$\begin{array}{l} \text{Effect }\left(\left(\%\right)\right)=\frac{\text{Average duration of loss of righting reflex in the test group}}{\text{Average duration of loss of righting reflex in the control}}\times 100 \end{array}$$

### Selection of compounds for pass prediction

Pedalitin, apigenin, methylgallate, 3,4-dihydroxybenzoic acid, calceolarioside, betonyoside A, campneoside II, acteoside, isoacteoside, and terniflorin were selected based on the availability as major compounds through literature survey (Rao et al., 2009). The structures of the compounds were collected from PubChem data base. Survey of quite a huge number of literatures made us to decide the above compounds as major compounds.

#### In silico experiment to predict the activity spectra for substances (PASS)

The selected phytoconstituents especially pedalitin, apigenin, methylgallate, 3,4-dihydroxybenzoic acid, calceolarioside, betonyoside A, campneoside II, acteoside, isoacteoside, terniflorin (Rao et al., 2009) were subjected for evaluating the antinociceptive activity with the aid of PASS program. This experiment predicts activity spectrum of a compound as probable activity (P_a_) and probable inactivity (P_i_) (Mohuya Mojumdar and Kabir, 2016) based on the structure-activity relationship analysis of the training set consisting of more than 205,000 compounds showing more than 3,750 types of biological activities. The values of Pa and Pi fluctuate between 0.000 and 1.000. A compound is considered experimentally active with Pa > Pi. Pa > 0.7, indicates the probability of pharmacological potential is high and the values following 0.5 < Pa < 0.7 reflect the considerable pharmacological effects experimentally. Pa < 0.5 shows less the pharmacological activity which may impart a chance of finding new compound (Goel et al., 2011; Khurana et al., 2011).

### *In silico* molecular docking

#### Preparation of protein

Three dimensional crystal structure of Cyclooxygenase-1 (COX 1, PDB id: 2OYE), cyclooxygenase-2 (COX 2, PDB id: 3HS5), and 5-HT1B (PDB id: 4IAQ) were downloaded in PDB format from the protein data bank (Berman et al., 2002). Structure was prepared and refined using the Protein Preparation Wizard of Schrödinger-Maestro v10.1. Charges and bond orders were assigned while hydrogens were added to the heavy atoms and selenomethionines were converted to methionines followed by deleting all water molecules. Using force field OPLS_2005, minimization was carried out setting maximum heavy atom RMSD (root-mean-square-deviation) to 0.30 Å.

#### Ligand preparation

Target compounds i.e., pedalitin, apigenin, methylgallate, 3,4-dihydroxybenzoic acid, calceolarioside, betonyoside A, campneoside II, acteoside, isoacteoside, and terniflorin were retrieved from Pubchem databases,. The 3D structures of the ligands were built by using Ligprep 2.5 in Schrödinger Suite 2015 with an OPLS_2005 force field. The pH 7.0 ± 2.0 was used to generate the ionization states of the compounds using Epik 2.2 in Schrödinger Suite. Up to 32 possible stereoisomers per ligand were retained.

#### Receptor grid generation

Receptor grids were calculated for prepared proteins so that various ligand poses bind within the predicted active site during docking. In Glide, grids were generated in a way to keep the default parameters of van der Waals scaling factor 1.00 and charge cutoff value 0.25 subjected to OPLS 2005 force field. A cubic box of specific dimensions centered on the centroid of the active site residues was generated for receptor. The bounding box was set to 14 × 14 × 14 × for docking experiments.

#### Glide standard precision (SP) ligand docking

SP flexible ligand docking was carried out in Glide of Schrödinger-Maestro v 10.1 (Friesner et al., 2004, 2006) within which penalties were applied to non-cis/trans amide bonds. For ligand atoms, Van der partial charge cutoff and scaling factor was selected to be 0.15 and 0.80, respectively. Final scoring was done on energy-minimized poses and showed as Glide score. The best docked pose with the lowest Glide score was recorded for each ligand.

### ADME/T property analysis

#### Ligand based ADME/toxicity prediction

The QikProp module of Schrodinger (Maestro, version 10.1) is a prompt, accurate, easy-to-use absorption, distribution, metabolism, and excretion (ADME) prediction program designed to produce certain descriptors linked to ADME. It predicts both pharmacokinetic and physicochemical significant descriptors relevant properties. ADME properties determine drug-like activity of ligand molecules based on Lipinski's rule of five. ADME/T properties of the compound (DIM) were analyzed using Qikprop 3.2 module (Natarajan et al., 2015).

### Statistical analysis

Data were presented as mean ± standard error of mean (SEM) values from triplicates. One-way analysis of variance (ANOVA) followed by Dunnet's test was used to describe the data for significant differences between the test and control groups using GraphPad Prism Data Editor for Windows, Version 6.0 (GraphPad software Inc., San Diego, CA). *P*-values (<0.05 and <0.01) were considered as statistically significant.

## Results

### Antinociceptive activities

#### Acetic acid induced writhing response

**Figure 1:**
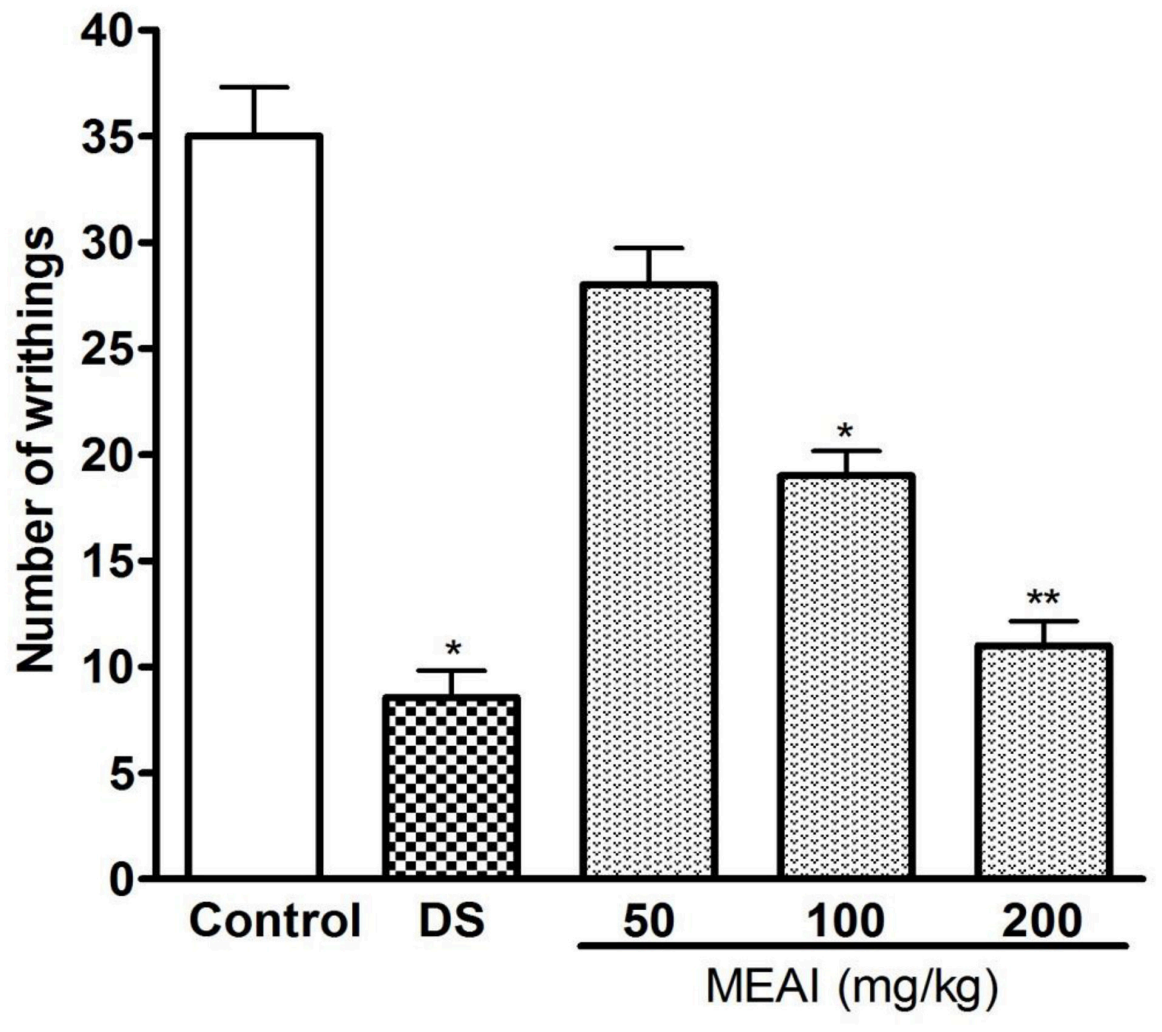
Effect of methanol extract of the *A. indica* and DS (10 mg/kg) on acetic acid induced writhing test. Values are mean ± S.E.M. ^*^p < 0.05 and ^**^p < 0.01, significantly different from control; ANOVA followed Dunnett's test (*n* = 6, per group). DS, diclofenac sodium; MEAI, methanolic extract of *A. indica*.

The results demonstrated the significant antinociceptive activity of MEAI as shown in Figure 1. The analgesic and writhing-inhibitory effect were increased with the dose in a noteworthy manner. The MEAI was found to be moderately active with its low dose and showed inhibitory effects of 20.00 and 45.71% at the doses of 50 mg/kg and 100 mg/kg, respectively. MEAI at a dose of 200 mg/kg exhibited the maximum inhibition of acetic acid induced writhing numbers which was significantly close (*p* < 0.05, *F* = 49.74) to that of the reference standard diclofenac sodium.

#### Formalin induced licking test

**Figure 2:**
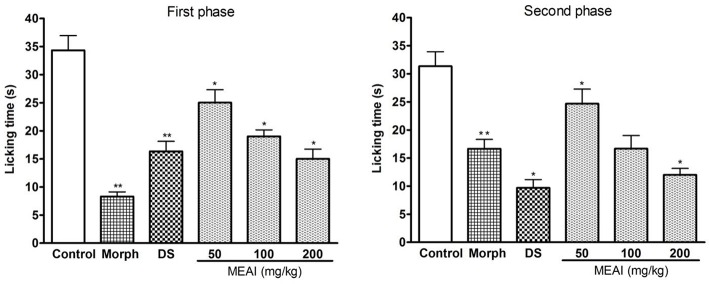
Effect of methanol extract of the *A. indica*, DS, and morphine (10 mg/kg) on formalin test (first phase and second phase). Values are mean ± S.E.M. ^*^p < 0.05 and ^**^p < 0.01, significantly different from control; ANOVA followed Dunnett's test (*n* = 6, per group). DS, diclofenac sodium; MEAI, methanolic extract of *A. indica*.

A characteristic biphasic nociceptive response is induced in the test animals after i.p administration of formalin. In both the phases the pain behaviors were evaluated individually by observing licking duration of animal models. The MEAI showed significant (*p* < 0.05) reduction of nociceptive behaviors evoked by formalin compared with control group. In the early/neurogenic phase (*F* = 24.03), MEAI showed protection percentages of 31.94, 45.18, and 58.53% at the doses of 50, 100, and 200 mg/kg, respectively. While in the late phase, MEAI markedly reduced the licking duration, significantly (*p* < 0.05, *F* = 15.63) lower than reference drug morphine, by exerting 44.18, 59.67, and 70.53% inhibition at the same concentration, respectively (Figure 2).

### Locomotor activity

#### Open field test

**Figure 3:**
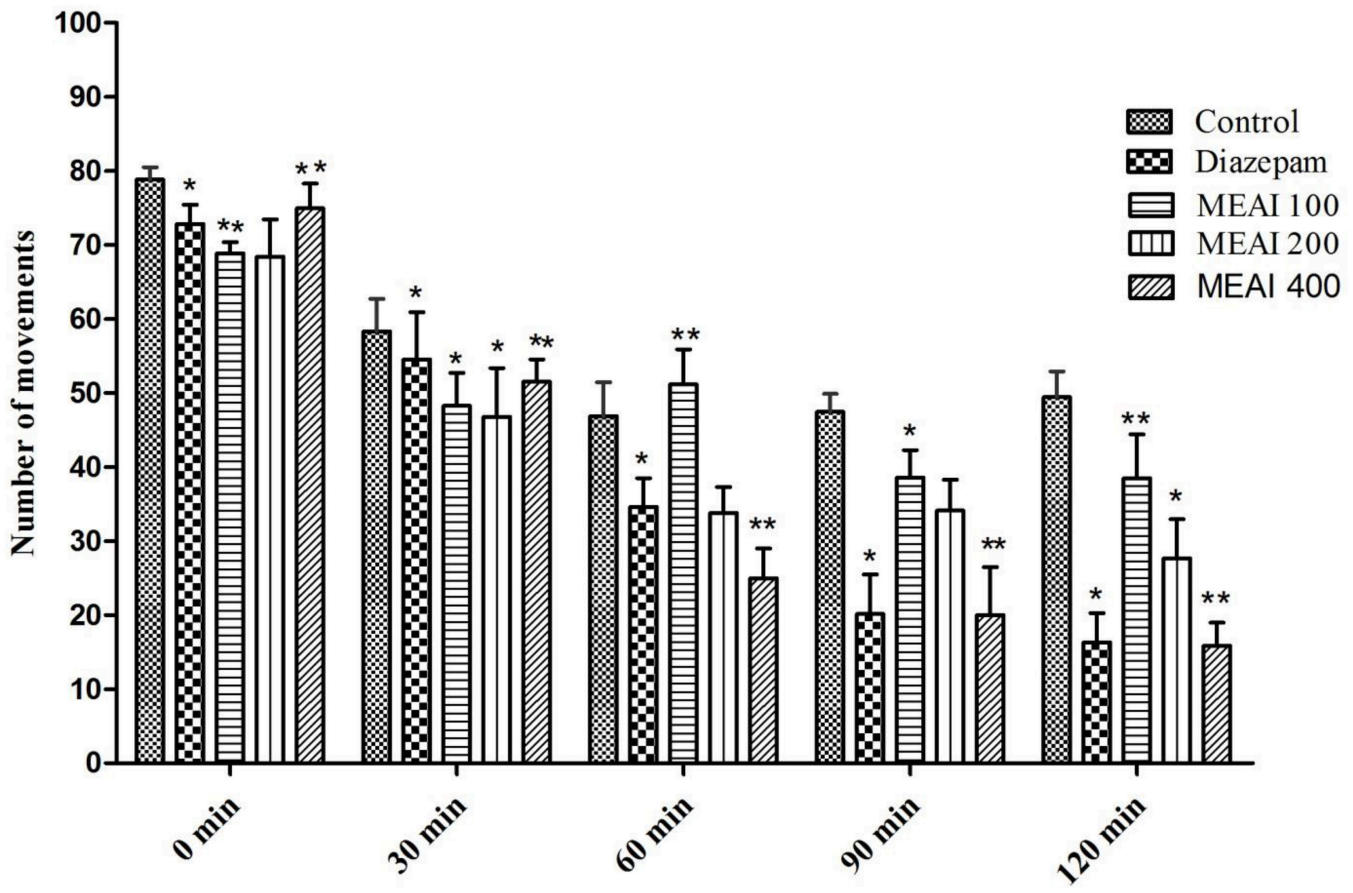
Effect of methanolic extract of *A. indica* on open field test in mice. Values are mean ± S.E.M. ^*^p < 0.05 and ^**^p < 0.01, significantly different from control; ANOVA followed Dunnett's test (*n* = 6, per group). MEAI, methanolic extract of *A. indica*.

The CNS activity of drug is evaluated by its effect on locomotion of the test animal model. In this test, the *A. indica* showed a dose dependent decrease in locomotor activity at the tested dose levels and the effects were statistically significant (*p* < 0.05). The locomotion lowering effect was pronouncedly evident from the second observation period continued till last observation period (120 min). In this study, test animal showed dose dependent decrease of movement figured as 38.52 ± 3.40, 27.71 ± 2.87, 15.90 ± 1.74 with 100, 200, and 400 mg/kg, respectively. While the reference drug diazepam (1 mg/kg) decreased (*p* < 0.05, *F* = 0.8140) the number of movement 54.56 ± 3.76, 34.59 ± 2.76, 20.15 ± 3.50, and 16.35 ± 3.12 with the similar doses from second observation period to last observation period (Figure 3).

#### Hole cross test

**Figure 4:**
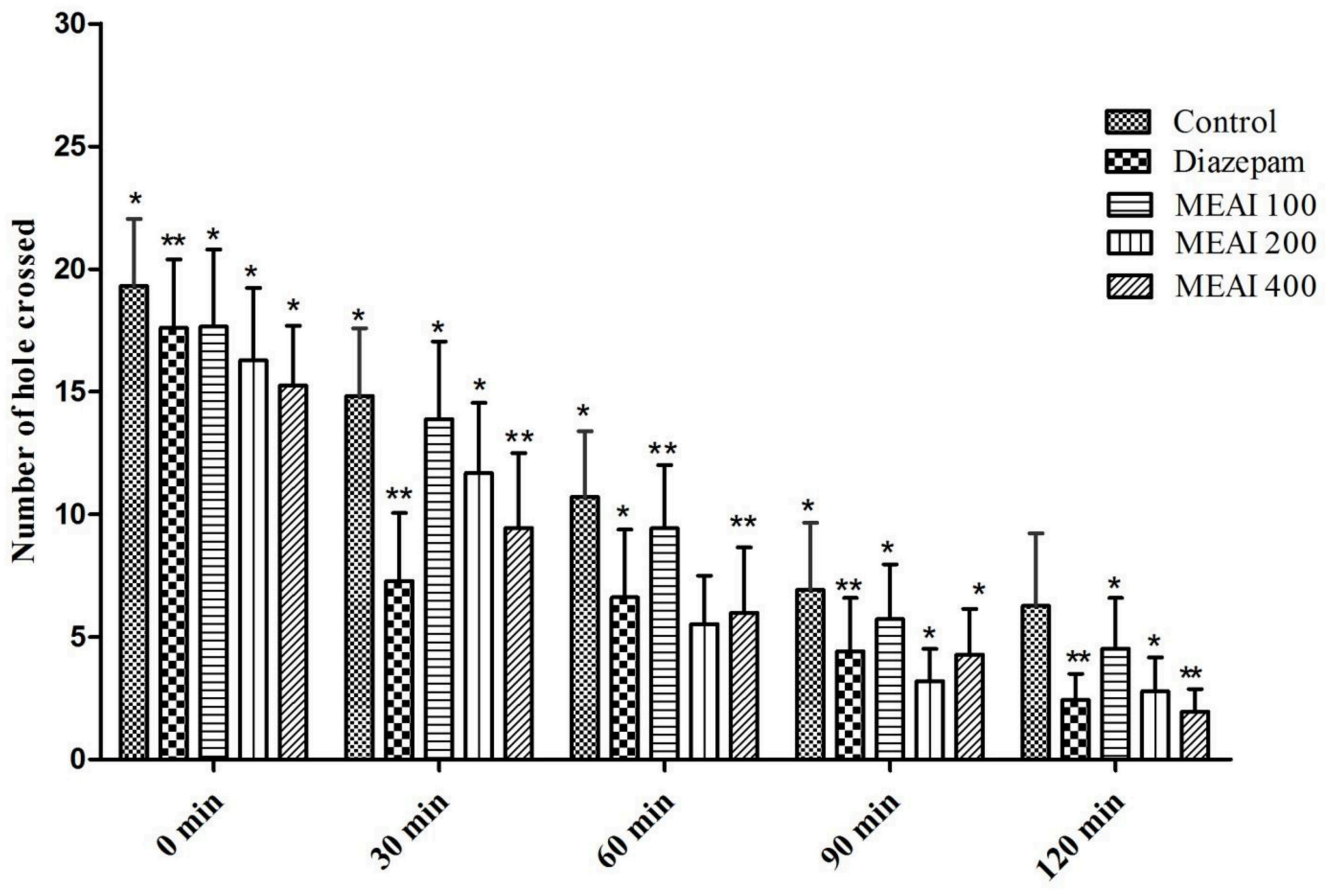
Effect of methanolic extract of *A. indica* on hole cross test in mice. Values are mean ± S.E.M. ^*^p < 0.05 and ^**^p < 0.01, significantly different from control; ANOVA followed Dunnett's test (*n* = 6, per group). MEAI, methanolic extract of *A. indica*.

MEAI demonstrated a gradual decrease in locomotion of the test animal model starting from second observation period (30 min) as confirmed by the reduction in the number of passes of tested mice through the hole in contrast to the control group (Figure 4). Therefore, a potent central nervous system (CNS) depressant activity was exhibited by MEAI at respective doses from second (30 min) to final (120 min) observation point which was statistically significant (*p* < 0.05–0.01). The results displayed the number of holes crossed at dose 400 mg/kg bw (1.95 ± 1.67) was comparable (*p* < 0.05, *F* = 0.5559) with the standard drug diazepam (2.78 ± 0.51) at final observation period (120 min). The other two dosages (100, 200 mg/kg bw) also reduced the locomotor activity (4.52 ± 1.21 and 2.10 ± 0.25, respectively). The CNS was found to be depressed during the observation period 0 to 120 min.

### Anxiolytic activity

#### Elevated plus-maze test (EPM)

**Figure 5:**
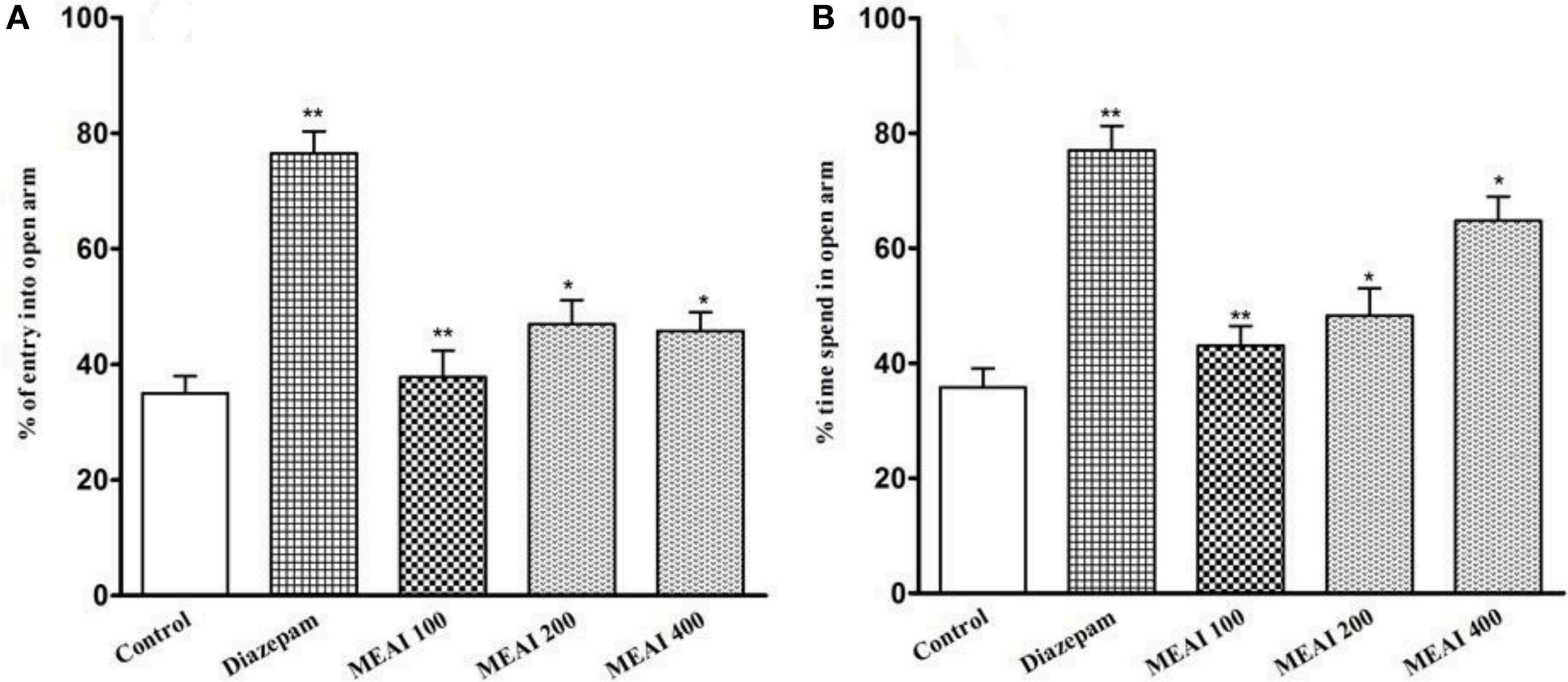
**(A)** Effect of methanolic extract of *A. indica* on percentage of entry into open arm in elevated plus maze test in mice. **(B)** Effect of methanolic extract of *A. indica* on percentage of time spent into open arm in elevated plus maze test in mice. Values are mean ± S.E.M. ^*^p < 0.05 and ^**^p < 0.01, significantly different from control; ANOVA followed Dunnett's test (*n* = 6, per group). MEAI: methanolic extract of *A. indica*.

As shown in Figure 5, all doses of MEAI increased the entries into the open arms. Meanwhile, the time spent into open arms was also increased to 43.04 ± 2.15, 48.25 ± 3.60, 64.75 ± 3.19 with the three doses. The values were statistically significant (*p* < 0.05, *F* = 18.81) compared to the control (35.75 ± 2.80). All the tested doses demonstrated a dose-dependent increase of time spent in open arms. Additionally, there was also a significant increase in duration of time (64.75 ± 3.19) for 400 mg/kg, into open arm, which was significant (*p* < 0.05, *F* = 17.52) in comparison to that (78.20 ± 4.12) of reference group.

### Sedative activity

#### Thiopental sodium induced sleeping time test

**Figure 6:**
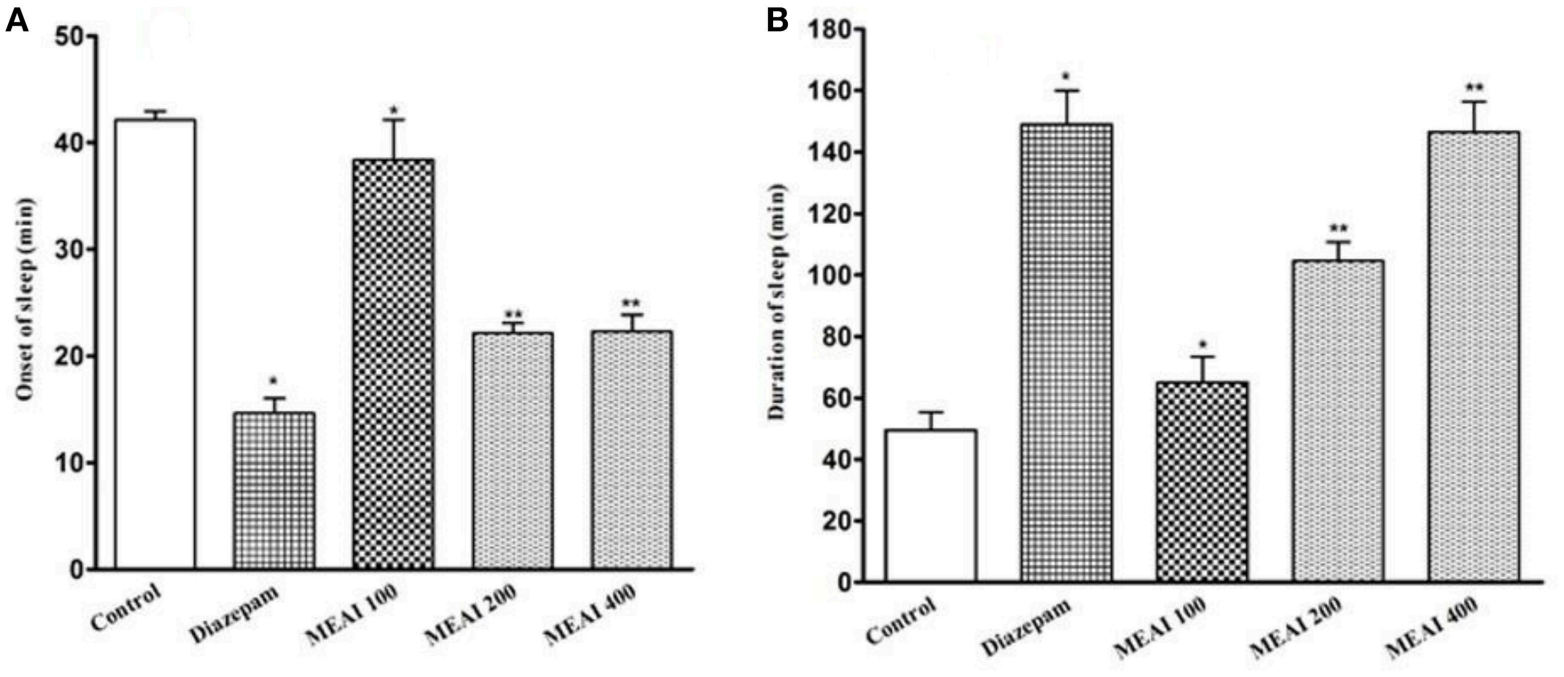
**(A)** Effect of methanolic extract of *A. indica* on onset of sleep in thiopental sodium induced sleeping time test in mice. **(B)** Effect of methanolic extract of *A. indica* on duration of sleep in thiopental sodium induced sleeping time test in mice. Values are mean ± S.E.M. ^*^p < 0.05 and ^**^p < 0.01, significantly different from control; ANOVA followed Dunnett's test (*n* = 6, per group). MEAI, methanolic extract of *A. indica*.

It was found that MEAI significantly (*p* < 0.05, *F* = 34.45) potentiated a decrease in onset of sleep and dose dependently increased in duration of sleep in thiopental-induced sleeping time test (*F* = 28.51). The prolongation of duration of sleeping time at dosages 100, 200, and 400 mg/kg (65.01 ± 4.71, 104.60 ±3.20, 146.46 ± 4.89, respectively) in comparison to control group (49.50 ± 2.70). Thiopental sodium induced sleeping time test was shown in Figure 6.

### *In silico* pass prediction

Ten constituents namely pedalitin, apigenin, methylgallate, 3,4-dihydroxybenzoic acid, calceolarioside, betonyoside A, campneoside II, acteoside, isoacteoside, terniflorin were analyzed by the PASS for their antinociceptive effects and results were used in a flexible manner. The chosen compounds showed higher P_a_ than P_i_ (Table 1). The compound 3,4-dihydroxybenzoic acid showed highest P_a_-value for antinociceptive activity (P_a_ = 0.563) followed by methylgallate (P_a_ = 0.537).

**Table 1:** Pass prediction of pedalitin, apigenin, methylgallate, 3,4-dihydroxybenzoic acid, calceolarioside, betonyoside A, campneoside II, acteoside, isoacteoside, terniflorin for antinociceptive activity.

**Phytocompounds with chemical structure**		**PASS prediction of antinociceptive activity**	
		**Pa**	**Pi**
Pedalitin	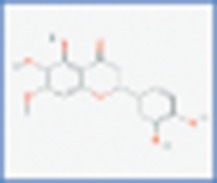	0.408	0.103
Apigenin	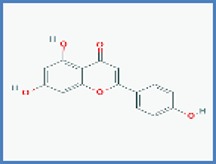	0.348	0.137
Methylgallate	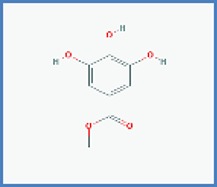	0.537	0.019
3,4-dihydroxybenzoic acid	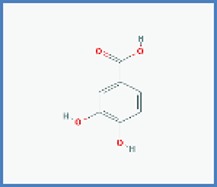	0.563	0.013
Calceolarioside	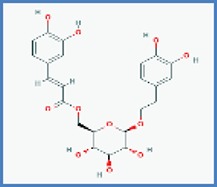	0.490	0.042
Betonyoside A	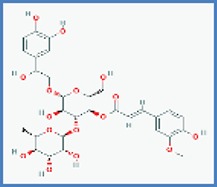	0.417	0.096
Campneoside II	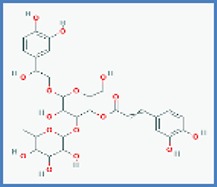	0.415	0.097
Acteoside	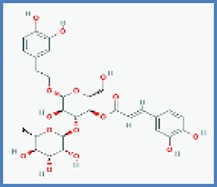	0.466	0.058
Isoacteoside	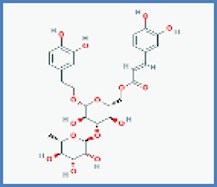	0.420	0.093
Terniflorin	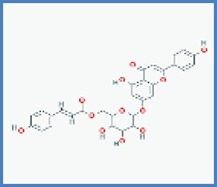	0.273	0.204

*Structures of the compounds have been take from PubChem*.

### *In silico* molecular docking analysis for analgesic effect

**Figure 7:**
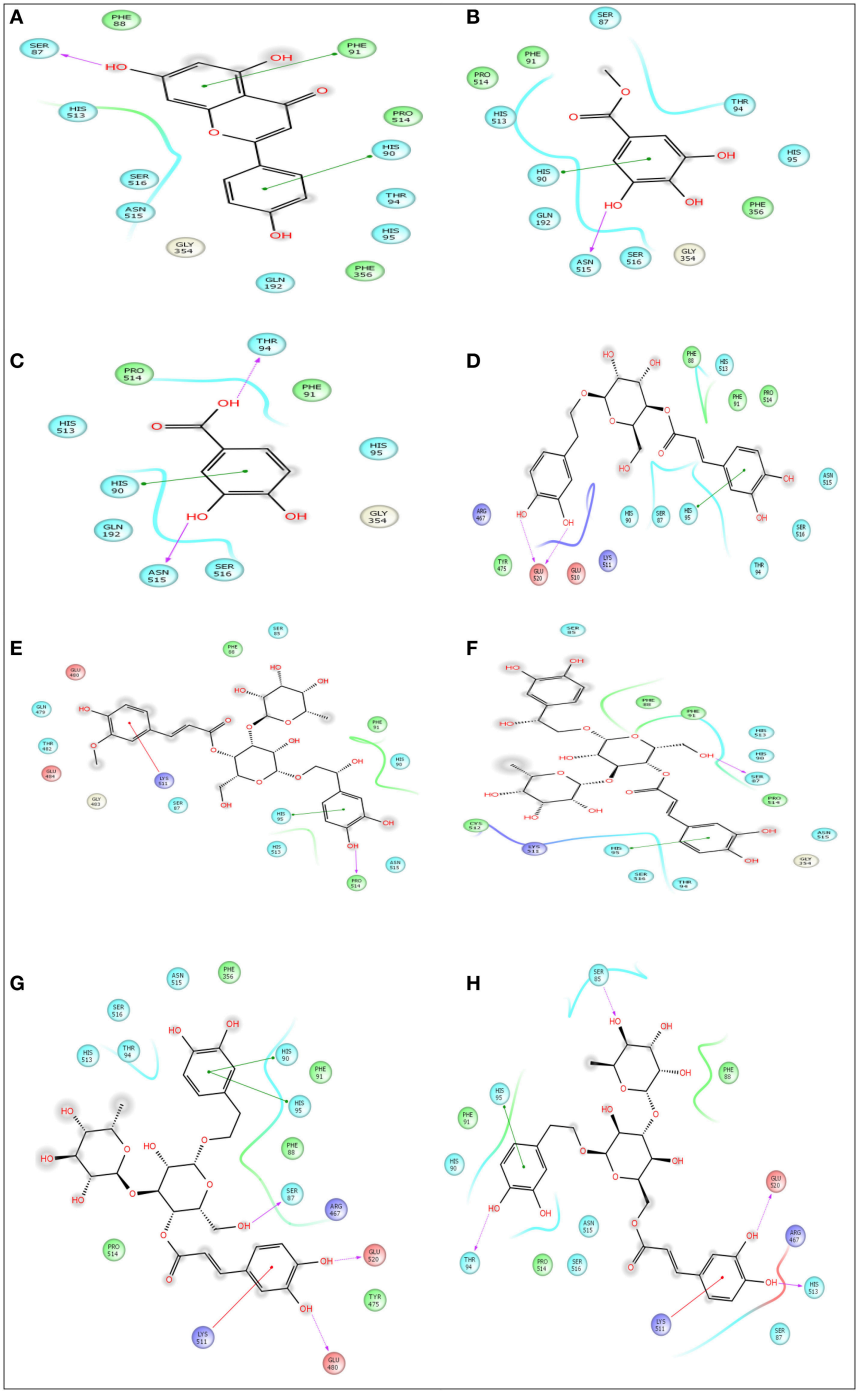
Docking results of apigenin **(A)**, methylgallate **(B)**, 3,4-dihydroxybenzoic acid **(C)**, calceolarioside **(D)**, betonyoside A **(E)**, campneoside II **(F)**, acteoside **(G)**, isoacteoside **(H)** with COX 1 (PDB: 2OYE) for analgesic effect. The colors indicate the residue (or species) type: Red-acidic (Asp, Glu), Green-hydrophobic (Ala, Val, Ile, Leu, Tyr, Phe, Trp, Met, Cys, Pro), Purple-basic (Hip, Lys, Arg), Blue-polar (Ser, Thr, Gln, Asn, His, Hie, Hid), Light gray-other (Gly, water), Darker gray-metal atoms. Interactions with the protein are marked with lines between ligand atoms and protein residues: Solid pink—H-bonds to the protein backbone, Dotted pink-H-bonds to protein side chains, Green—pi-pi stacking interactions, Orange-pi-cation interactions. Ligand atoms that are exposed to solvent are marked with gray spheres. The protein “pocket” is displayed with a line around the ligand, colored with the color of the nearest protein residue. The gap in the line shows the opening of the pocket.

**Figure 8:**
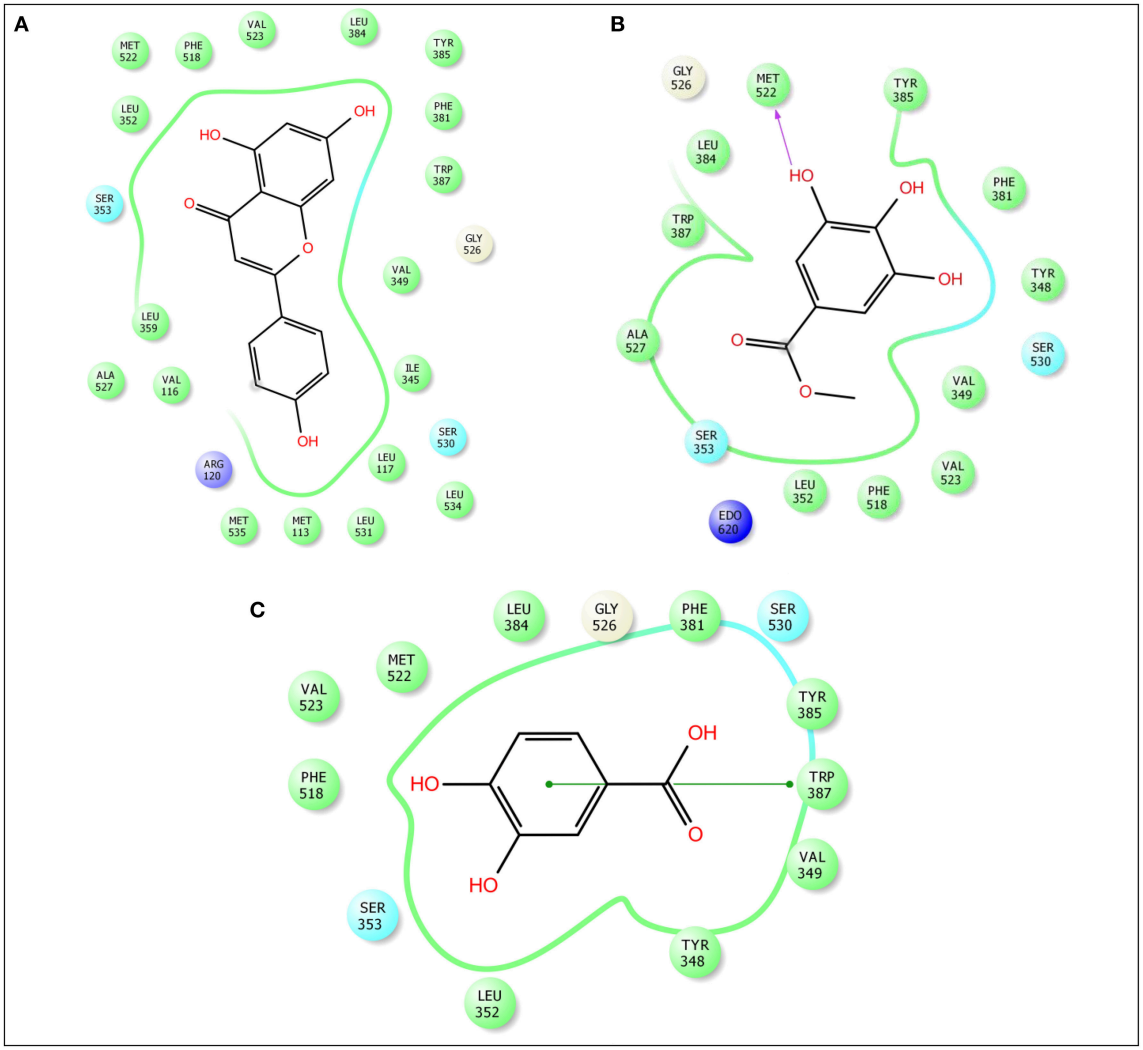
Docking results of apigenin **(A)**, methylgallate **(B)**, 3,4-dihydroxybenzoic acid **(C)** with COX 2 (PDB: 3HS5) for analgesic effect. The colors indicate the residue (or species) type: Red-acidic (Asp, Glu), Green-hydrophobic (Ala, Val, Ile, Leu, Tyr, Phe, Trp, Met, Cys, Pro), Purple-basic (Hip, Lys, Arg), Blue-polar (Ser, Thr, Gln, Asn, His, Hie, Hid), Light gray-other (Gly, water), Darker gray-metal atoms. Interactions with the protein are marked with lines between ligand atoms and protein residues: Dotted pink-H-bonds to protein side chains, Green—pi-pi stacking interactions, Orange-pi-cation interactions. Ligand atoms that are exposed to solvent are marked with gray spheres. The protein “pocket” is displayed with a line around the ligand, colored with the color of the nearest protein residue. The gap in the line shows the opening of the pocket.

**Figure 9:**
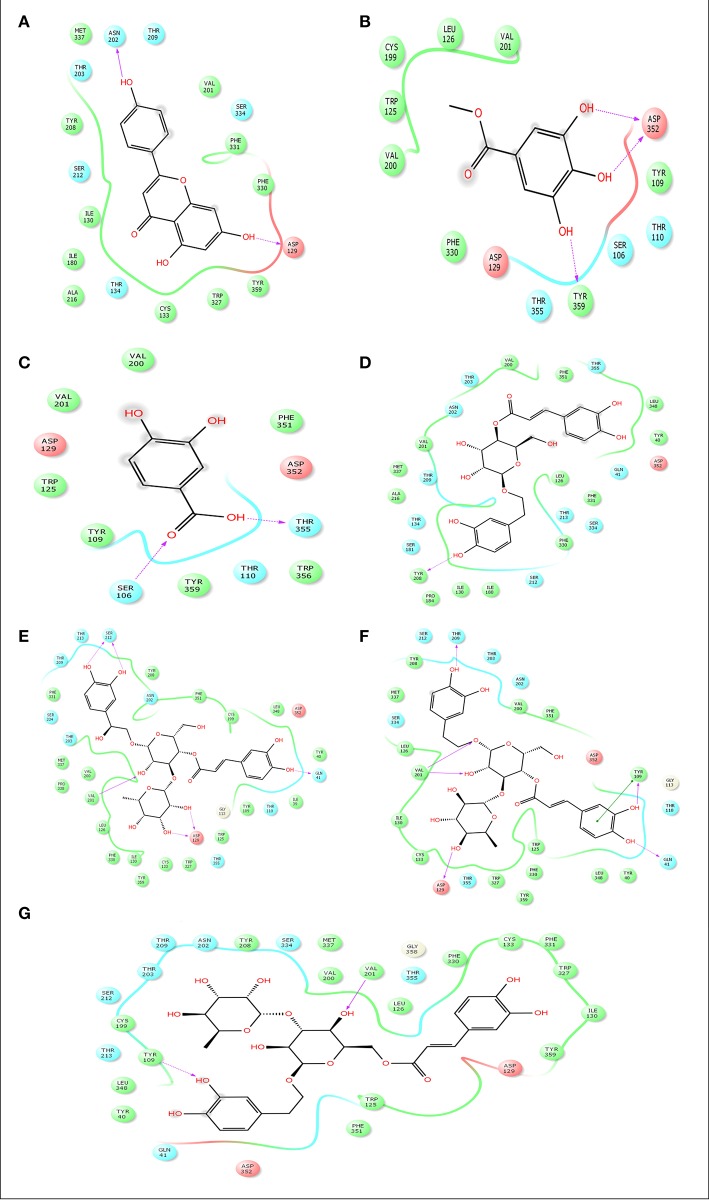
Docking results of apigenin **(A)**, methylgallate **(B)**, 3,4-dihydroxybenzoic acid **(B)**, calceolarioside **(D)**, campneoside II **(E)**, acteoside **(F)**, isoacteoside **(G)** with 5-HT1B (PDB id: 4IAQ) for antidepressant effect. The colors indicate the residue (or species) type: Red-acidic (Asp, Glu), Green-hydrophobic (Ala, Val, Ile, Leu, Tyr, Phe, Trp, Met, Cys, Pro), Purple-basic (Hip, Lys, Arg), Blue-polar (Ser, Thr, Gln, Asn, His, Hie, Hid), Light gray-other (Gly, water), Darker gray-metal atoms. Interactions with the protein are marked with lines between ligand atoms and protein residues: Solid pink—H-bonds to the protein backbone, Dotted pink-H-bonds to protein side chains, Green—pi-pi stacking interactions, Orange-pi-cation interactions. Ligand atoms that are exposed to solvent are marked with gray spheres. The protein “pocket” is displayed with a line around the ligand, colored with the color of the nearest protein residue. The gap in the line shows the opening of the pocket.

Advancement and sophistication of computational techniques have developed virtual screening to get a positive impact on the process of discovery. Virtual screening process uses the scoring and docking of the compound individually from a database while the fundamental of the technique is prediction of binding modes and affinity of the compounds by means of docking to an X-ray crystallographic structure. Grid based docking model was used to analyze the binding pattern of molecules with the amino acids present in the active pocket of the protein. To evaluate the potential analgesic molecule, we have undertaken the docking analysis of the isolated active compounds of *A. indica* to the active site cyclooxygenase enzymes viz. COX-1. Fourteen compounds so far isolated by different researchers from A. *indicia* have been listed up (Rao et al., 2009) and the prevailing 10 compounds have been chosen to find out the most potential compounds suitable for therapeutic approaches through *in silico* docking study. Despite the diverse chemical structures of non-steroidal anti-inflammatory drugs, their analgesic effects are mainly due to the common property of inhibiting cyclo-oxygenases (COX) involved in the formation of prostaglandins, the inflammatory mediators, which are formed by the conversion of arachidonic acid. Therefore, this study has chosen the isoforms Cox-1 and Cox-2 of Cox enzyme for *in silico* model (Lenardão et al., 2016). Additionally the receptor 5-HT1B was selected as the receptor as well modulates the antinociception through its agonist binding (Jeong et al., 2012). For studying the interaction of the compounds acteoside, betonyoside A, β-sitosterol, isoacteoside, stigmasterol with 2OYE,Glide docking analysis was performed by Schrodinger suite v10.1, where among of these compounds apigenin shows highest docking score shown in Table 2. The low and negative free energy values for binding indicates a strong favorable bond between 2OYE. According to Docking Score, apigenin was found to have the highest affinity to the COX-1 enzymes corresponding to the methylgallate, 3,4-dihydroxybenzoic acid and calceolarioside. The results of docking analysis were presented in Tables 2–4 and the docking figure showed in Figures 7–9.

**Table 2:** Docking results of pedalitin, apigenin, methylgallate, 3,4-dihydroxybenzoic acid, calceolarioside, betonyoside A, campneoside II, acteoside, isoacteoside, terniflorin with COX 1 (PDB: 2OYE) for analgesic effect.

**Compound name**	**Docking score**	**Glide e model**	**Glide energy**
Apigenin	−6.558	−45.804	−33.285
Methylgallate	−5.568	−36.628	−27.553
3,4-dihydroxybenzoic acid	−5.836	−36.615	−28.943
Calceolarioside	−3.944	−43.087	−38.449
Betonyoside A	−2.468	−27.687	−26.796
Campneoside II	−4.711	−47.817	−40.214
Acteoside	−4.232	−43.177	−37.819
Isoacteoside	−3.196	−16.98	−12.443
Pedalitin	–	–	–
Terniflorin	–	–	–

**Table 3:** Docking results of apigenin, methylgallate, 3,4-dihydroxybenzoic acid with COX 2 (PDB: 3HS5) for analgesic effect.

**Compound name**	**Docking score**	**Glide e model**	**Glide energy**
Apigenin	−8.441	−59.92	−40.316
3,4-dihydroxybenzoic acid	−5.18	−26.881	−18.894
Methylgallate	−6.303	−40.433	−29.26

**Table 4:** Docking results of apigenin, methylgallate, 3,4-dihydroxybenzoic acid, calceolarioside, campneoside II, acteoside, isoacteosidewith 5-HT1B (PDB id: 4IAQ) for antidepressant effect.

**Compound name**	**Docking score**	**Glide e model**	**Glide energy**
Apigenin	−7.584	−62.57	−42.95
Methylgallate	−5.246	−40.13	−30.777
3,4-Dihydroxybenzoic acid	−4.763	−32.943	−26.42
Calceolarioside	−5.263	−49.171	−34.29
Campneoside II	−6.279	−50.314	−33.303
Acteoside	−6.094	−48.583	−37.06
Isoacteoside	−5.227	−62.228	−48.055

### Toxicity and ADME analysis through ligand based toxicity/ADME prediction

The drug-like activity of the ligand molecule was classified using ADME properties by QikProp module of Schrodinger. The ADME properties of the pedalitin, apigenin, methylgallate, 3,4-dihydroxybenzoic acid, calceolarioside, betonyoside A, campneoside II, acteoside, isoacteoside, terniflorin were clarified with QikProp module of Schrodinger, shown in Table 5. The selected properties are known to influence cell permeation, metabolism, and bioavailability. Predicted properties of the pedalitin, apigenin, methylgallate, 3,4-dihydroxybenzoic acid were in the range to satisfy the Lipinski's rule of five to be recognized as drug like potential. All other compounds were not satisfying the all five rules.

**Table 5:** ADME/T properties of pedalitin, apigenin, methylgallate, 3,4-dihydroxybenzoic acid, calceolarioside, betonyoside A, campneoside II, acteoside, isoacteoside, terniflorin by QikProp.

**Name of molecules**	**Pubchem ID**	**MW^α^**	**HB donor^β^**	**HB acceptor^e^**	**Log P^¥^**	**Molar refractivity^μ^**
Pedalitin	31161	316.26	4	7	2.02	78.41
Apigenin	5280443	270.0	0	5	1.49	61.37
Methyl gallate	7428	184.15	3	5	0.85	43.67
3,4- dihydroxy-benzoic acid	72	154.12	3	4	0.88	36.94
Calceolarioside	5273566	478.45	7	11	0.47	116.21
Betonyoside A	102000760	654.61	9	16	−1.13	154.75
Campneoside II	85091108	640.59	10	16	−1.44	149.91
Acteoside	5281800	624.59	9	15	−0.45	148.40
Isoacteoside	6476333	624.59	9	15	−0.23	148.40
Terniflorin	6439941	578.52	6	12	3.04	145.89

α *Molecular weight (acceptable range: <500)*.

β *Hydrogen bond donor (acceptable range: ≤ 5)*.

e *Hydrogen bond acceptor (acceptable range: ≤ 10)*.

¥ *High lipophilicity (expressed as LogP, acceptable range: <5)*.

μ *Molar refractivity should be between 40 and 130*.

## Discussion

Pain is one of the most pervasive problems in current time and it has high social and economic impacts (Julius and Basbaum, 2001). During inflammation, several mediators can activate and/or sensitize nociceptive fibers and mediators; these mediators are also involved in edema formation and leukocyte infiltration (Mazzon et al., 2008). Several analgesics are used to treat a wide range of painful and inflammatory conditions, including non-steroidal anti-inflammatory drugs (NSAIDs), glucocorticoids and opioids (Ferreira et al., 1997). Despite the high diversity of available anti-inflammatory and analgesic drugs, their side effects and the ineffectiveness of some drugs for some conditions require the continuous search for new drugs. Investigating plant–derived products is a vital way to discover effective and less toxic drugs (Fine et al., 2009).

This study therefore investigated the anti-nociceptive activity of MEAI in classical non-narcotic peripheral and central acting pain models by acetic acid induced writhing test and formalin induced licking test, respectively. Acetic acid induced writhing test evaluated the antinociceptive activity of MEAI characterizing through abdominal contractions, body movements as a whole and twisting of the abdominal muscles. This non-specific pain evaluating method is suitable to detect the effects showed by weak analgesics. Pain is generated by endogenous inflammatory mediators such as serotonin and bradykinin which stimulate peripheral nociceptive neurons (Sakiyama et al., 2008). The pain-sensation in acetic acid induced model is triggered by localized inflammatory response for the release of free arachidonic acid from tissue phospholipids via cyclooxygenase (COX-1 and COX-2), and prostaglandin specifically PGE2 and PGF2 biosynthesis, the level of lipoxygenase products may also be increased in peritoneal fluids (Adzu et al., 2003). MEAI administration significantly reduced acetic acid-induced writhing in mice. This result supports the hypothesis that the extract from *A. indica* may act by inhibiting prostaglandin synthesis because the nociceptive mechanism of abdominal writhing induced by acetic acid involves the release of arachidonic acid metabolites via cyclooxygenase (COX), and prostaglandin biosynthesis. Additionally, the possible involvement of neurotransmitter systems, such as opioid, serotonergic, purinergic, cholinergic, catecholaminergic, cannabinoid, GABAergic systems as well as ATP-gated potassium channels could be involved. Apart from these, flavonoid and their derivatives have been found to be antinociceptive and anti-inflammatory agents due to their ability to inhibit arachidonic acid metabolism (Middleton et al., 2000). It is possible that the presence of apigenin, terniflorin and other flavonoid molecules in the extract of *A. indica* be responsible for the antinociceptive effect. *In silico* study also complies with the binding of cyclooxygenase with the target compounds especially 3,4-dihydroxybenzoic acid and apigenin used in this study.

The formalin test is one of the widely used methods of expressing pain and analgesic mechanism in contrast to mechanical or thermal stimulus methods (Khan et al., 2010). Applying this method we can also differentiate between the peripheral and central antinociceptive pain. This is a biphasic model where the early phase represents neurogenic (1–5 min) and late phase represents inflammatory pain (15–30 min), respectively (Dallel et al., 1995). The early phase demonstrates an acute response observed immediately after the administration of formalin because of direct stimulation of nociceptive neurons. While the late phase gives a delayed response made by the release of inflammatory mediators especially prostaglandins, histamine, serotonin and bradykinin, and activation of the neurons in the dorsal horns of the spinal cord (Clavelou et al., 1995). Administration of MEAI in different doses significantly inhibited the pain response in both phases as confirmed by reduced licking behavior but the effect was more prominent in the late phase. Considering the inhibitory property of MEAI on the second phase of formalin, we might suggest an anti-inflammatory action of the plant extract. This result is in line with that obtained in formalin model and indicates that MEAI probably interacts with COX receptors occupied by the *A. indica* compounds showing formalin-induced nociception. Opioid analgesics exert its antinociceptive effects for both phases while the early phase is more sensitive whereas NSAIDs seem to suppress only the late phase. Therefore, reduced liking time in both phases indicates a possible interaction with neurogenic and inflammatory pain modulators.

To evaluate the drug action on CNS, observation of locomotor activity of the test animal is regarded as a noteworthy method. Because this activity is regarded as an indicator of alertness and a decreased locomotor performance has been used as an index of CNS depressant activity (Hunskaar and Hole, 1987). Various researches have confirmed that open field method is a classic model to evaluate general and spontaneous activity of animals (Sousa et al., 2004; Gahlot et al., 2013). The decrease in locomotor activity due to the treatment with MEAI in the tested mice was corresponding with the effect of antidepressants because sensory afferent neurons provide excitatory feedback to motor neurons and spinal interneurons in response to muscle contraction during active locomotion. Additionally local GABAergic interneurons modulate this pathway by inhibiting sensory afferents at the presynaptic level (Rudomin, 2009). Pharmacological manipulations showed that GABAergic neurons modulate the burst frequency of motor neurons during fictive locomotion (Schmitt et al., 2004).

The hole-cross test is similar to the open field test in the constructs that it can assess locomotor activity and detect the wide spectrum antidepressants. Accordingly, it is believed that complementary and/or converging information on potential antidepressants could be achieved by using both the tests can. From our study data, it is evident that i.p. administration of MEAI significantly suppressed the spontaneous locomotion of test mice in the hole-cross test. Substances which have sedative and CNS depressant activity either decrease the time for onset of sleep or prolong the duration of sleep or both. It is possible that MEAI act by potentiating GABAergic inhibition in the CNS via membrane hyperpolarization since GABA is the major inhibitory neurotransmitter in the CNS and it leads to a reduction in the firing rate of critical neurons in the brain. This inhibition may be due to direct activation of GABA receptor by MEAI. It may also be due to enhanced affinity for GABA or an increase in the duration of the GABA-gated channel opening (Barria et al., 1997).

The use of the elevated plus maze (EPM) test is considered to be better examine anxiety-like behavior (Katz et al., 1981). Drugs having anxiolytic activity decreases distance for open arms and induces test animal to spend much time in open arms whereas anxiogenics decrease open arm exploration by reducing both the number of entries and spent time into the open arms (Griebel et al., 2000). Administration of MEAI displayed an inclination of increasing the time spent in the open arms, an indicator of decreased anxiety implying a reduction in anxiety-like behavior. It is believed that antidepressive efficiency in the elevated plus-maze test is a bit controversial because acute antidepressants may give rise to anxiogenic effect in this paradigm (Kõks et al., 2001; Holmes and Rodgers, 2003; Drapier et al., 2007). However, anxiety behavior was not induced by MEAI.

Thiopental sodium induced sleeping test was used for assessing the sleeping behavior. Indeed, thiopental induces hypnosis by potentiating GABA mediated post synaptic inhibition caused due to the allosteric modification of GABA_A_ receptors. Components possessing CNS depressant effects either reduce the time of onset of sleep or extend the sleep duration or both (Nyeem et al., 2006; Hasan et al., 2009). Earlier, it has been found a relationship between enhancement of hypnosis and index of CNS depressant activity (Fujimori, 1965). Phytochemical analysis revealed that leaves of *A. indica* contain alkaloids and tannins as major phytochemicals along with saponins, glycosides, carotenoids, polyuronoides and aromatic oil as bioactive constituents (Ulhe and Narkhede, 2013). Moreover, several behavioral researches demonstrated that isoquinoline-type alkaloid structures possess anticonvulsant and hypnotic properties (Singla et al., 2010). The presence of alkaloidal phytochemicals in *A. indica* may exert its sedative activity possibly through inhibition of GABA_A_ receptors.

*A. indica* containing flavonoids, terpenoids, and propanoids exerted anti-inflammatory activities, *in vitro* experiment displayed previously (Rao et al., 2009). Further support for the significance of our results is the finding of antinociceptive, antidepressive, and anxiolytic properties of MEAI *in vivo*. Earlier, it has been reported that pathophysiology of major depression is associated with oxidative stress (Bilici et al., 2001). Therefore, treatment with antioxidant may reduce the oxidative stress and depressive disorder as well. Recently, we have reported that *A. indica* possesses anticholinesterase and antioxidative properties (Uddin et al., 2016)which may substantiate the possible contribution to its antidepressant-like effect.

PASS computer program was used to screen biological activity of selected compounds as antinociceptive. The compound 3,4-dihydroxybenzoic acid showed the highest P_a_-value (P_a_ = 0.563) for antinociceptive activity which was preceded by methyl gallate showed the P_a_-value 0.537 indicating a high probability of these compounds acting as antinociceptive agents. Previous researches showed some benzoic acid derivatives to be involved in the antinociceptive action with direct or indirect activation of opioid receptors (Déciga-Campos et al., 2007).

This research has conducted a molecular docking analysis, which allows accurate prediction of ligand and receptor interaction as well as binding energy, to have a good picture of Cyclooxygenase *A. indica* compounds interaction. Grid based docking study was used to analyze the binding modes of molecules with the amino acids present in the active pocket of the protein (Veeramachaneni et al., 2015). To identify a potential lead molecules for analgesic activity and antidepressant effect, docking analysis of the active compounds *A. indica* has been performed with the active site of cyclooxygenase enzymes-1 (COX-1), cyclooxygenase enzymes-2 (COX-2) and serotonin receptors. The interaction between compounds and the active site was assessed with docking analysis by Schrodinger suite v10.1.

Among of the compounds of *A. indica*, apigenin gives the lowest docking score −6.558 and −8.441 with COX-1 and COX-2 receptors, respectively, followed by methylgallate and 3,4-dihydroxybenzoic acid. It has been evident that the negative and low binding energy demonstrates a strong bonding. Additionally, apigenin is the potential compound for non-selective analgesic activity. The antinociceptive and neuroprotective effect of this flavonoid molecule is already been established (Kowalski et al., 2005).

In addition, the performance of molecular docking, determining the antidepressant activity among of the isolated compounds with the serotonin receptors, apigenin displayed the negative and low value (−7.584) of free energy of binding demonstrates making a strong favorable bond. Docking score suggests apigenin might be the responsible compound for potential antidepressant activity.

Virtual screening is important for natural product chemists to search the theoretically active principles with attractive ADME/T profiles which have been isolated previously but not assayed the activity against specified drug targets (Ntie-Kang et al., 2013). This prediction procedure can be a better option for lead search than the random screening. From the results of ADME/T test, it is clear that among all the compounds only pedalitin, apigenin, methylgallate, and 3,4-dihydroxybenzoic acid were in the range for satisfying the Lipinski's rule of five to be considered as potential drug in terms of better pharmacokinetics properties with less toxicity.

## Conclusion

The methanol extract of all tested doses of *A. indica* possesses analgesic activity in central as well as peripheral pain models. In addition, results of the present neuropharmacological study of *A. indica* exhibited antidepressant and anxiolytic activity with less sedative side effects. In both cases the effect was dose dependent and statistically significant. Moreover, the computer programs PASS reflected the antinociceptive activity; molecular docking demonstrated higher binding affinity with COX-1, COX-2, serotonin and ADME/Toxicity analysis displayed the satisfactory pharmacokinetics and toxicity profiles of 3,4-dihydroxybenzoic acid and apigenin. Further research must be conducted to elucidate the antinociceptive and neuropharmacological activities of selected potential compounds in animal model with a dose-response study.

## Author contributions

MU, AA, and MA-A-M together planned and designed the research and arrange the whole facilities for the research; MN, SA, and MA conducted all the laboratory works, MK performed the *in silico* works using bioinformatics tools, MR imparted in study design and interpreted the results putting efforts on statistical analysis with MU, AA, and MA-A-M.

### Conflict of interest statement

The authors declare that the research was conducted in the absence of any commercial or financial relationships that could be construed as a potential conflict of interest. The reviewer ND and handling Editor declared their shared affiliation.
